# The epigenome as a putative target for skin repair: the HDAC inhibitor Trichostatin A modulates myeloid progenitor plasticity and behavior and improves wound healing

**DOI:** 10.1186/s12967-019-1998-9

**Published:** 2019-07-31

**Authors:** Mariana Cabanel, Thayse Pinheiro da Costa, Marcia Cury EL-Cheikh, Katia Carneiro

**Affiliations:** Laboratório de Proliferação e Diferenciação Celular, Universidade Federal do Rio de Janeiro, Rio de Janeiro, Brazil

**Keywords:** Histone deacetylase, Bone marrow myeloid progenitors, Macrophage plasticity, Wound healing

## Abstract

**Background:**

The molecular pathways that drive bone marrow myeloid progenitors (BMMP) development are very well understood and include a tight controlled multi-stage gene hierarch. Monocytes are versatile cells that display remarkable plasticity and may give rise to specific subsets of macrophages to proper promote tissue homesostasis upon an injury. However, the epigenetic mechanisms that underlie monocyte differentiation into the pro-inflammatory Ly6C^high^ or the repairing Ly6C^low^ subsets are yet to be elucidated. We have previously shown that Epigenetic mechanisms Histone Deacetylase (HDAC) dependent are crucial for monocyte behavior and plasticity and in this work, we propose that this same mechanism underlies BMMP plasticity upon an inflammatory challenge in vivo.

**Methods:**

BMMP were culture in the presence of GM-CSF alone or in combination with HDAC inhibitor (iHDAC) and phenotyped by flow cytometry, immune staining or western blot. iHDAC was topically added to skin wounds for 7 consecutive days and wound healing was monitored by flow cytometry and histopathological analysis.

**Results:**

When BMMP were cultured in the presence of iHDAC, we showed that the CD11b^low^/Ly6C^low^ subset was the specific target of iHDAC that underwent chromatin hyperacetylation in vitro. Upon 13 days in the presence of iHDAC, BMMP gave rise to very elongated macrophages, that in turn, displayed a remarkable plasticity in a HDAC activity dependent fashion. HDAC-dependent cell shape was tight related to macrophage behavior and phenotype through the control of iNOS protein levels, showing that chromatin remodeling is a key component of macrophage plasticity and function. We then hypothesized that iHDAC would modulate the inflammatory response and favor tissue repair in vivo. To test this hypothesis, we topically added iHDAC to skin wounds during 7 consecutive days and followed tissue repair dynamics. In fact, iHDAC treated skin wounds presented an increase in wound closure at day 5 that was correlated to an enrichment in the CD11b^low^/Ly6C^low^ subset and in very elongated F4/80 positives macrophages in vivo, fully recapitulating the behavior previously observed in vitro.

**Conclusion:**

Our work provides the biological basis that connects chromatin remodeling to phenotypic plasticity, which in turn, may become a tractable therapeutic strategy in further translational studies.

**Electronic supplementary material:**

The online version of this article (10.1186/s12967-019-1998-9) contains supplementary material, which is available to authorized users.

## Background

Modulation of the inflammatory response is essential for its resolution and for the return of the system to homeostasis. As key players in this modulation, we highlight the cells of the innate system of myeloid origin. In the adult, these cells originate from definitive hematopoietic stem cell (HSC) through a well characterized differentiation program upon control of specific growths factors and stromal cells in the bone marrow environment [1, 2]. This network of interactions leads common myeloid progenitors cells (CMPs) to give rise to committed granulocyte-macrophage progenitors (GMP), which in turn, will progressively generate granulocytes and monocyte-dendritic progenitors (MDP) with potential to differentiate, at least, in classical dendritic cells (cDC) and in specific monocytes progenitors (MNp) [1, 3]. In both, granulocytes and monocytes progenitors, the expression of CD11b/CD18 (α[M]β2 integrin) and Ly6C (a GPI-linked cell surface antigen) are up or down modulated along the cascade of myeloid cells differentiation in the presence of GM-CSF (Granulocyte and Macrophage Colony Stimulating Factor). Thus, the CMP give rise to different sub sets characterized as: CD11b^hi^/Ly6C^low^, majority composed by granulocytes, and Ly6C^high^ and Ly6C^low^ sub population, both expressing CD11b^low^, composing the MNp pool [1, 3]. Thus, the myeloid progenitors are a large and heterogeneous cell population harboring mainly a CD11b and Ly6C markers which are differentially expressed along the terminal cascade of cell differentiation with specific phenotype and functions.

In mice, these cells are involved in defense against pathogens [4] as well as in mechanisms of tissue repair and wound healing [5]. These cells may have a Ly6C^high^ phenotype and are meant to be inflammatory cells which are rapidly recruited to injury or infection sites and give rise to terminally differentiated pro-inflammatory M1 macrophages [6]. Ly6C^low^ monocytes are involved in peripheral patrol of blood vessels and have been implicated in promoting wound healing and tissue repair (M2 macrophages) [7, 8]. They are capable of infiltration and differentiation in F4/80^+^ tissue macrophages and can secrete a range of cytokine and chemokines, actively participating in the process of clearance and resolution of inflammation. It is widely discussed in the literature whether such cells are able to differentiate from individual precursors or whether they can undergo switch between M1/M2 phenotypes if properly stimulated by trophic factors, cytokines and chemokines [9]. However, their plasticity is as undeniable as essential for them to perform versatile and dynamic functions in the face of a wide range of environmental cues [10]. Far beyond a simple dichotomy we can clearly perceive the broad spectrum of phenotypic and functional possibilities of these cells [11]. However, while well documented, it is still unclear how such incredible versatility arises and what are the mechanisms involved in this process.

In fact, we have recently demonstrated that HDAC activity is required to confer plasticity to myeloid progenitors even in the presence of pro-inflammatory cues [12]. Notably, when HDAC activity was blocked during bone marrow myeloid precursors (BMMP) differentiation in vitro, macrophages differentiated from such progenitors presented an elongated morphology and harbored a mixed phenotype of the M1/M2 even in the presence of GM-CSF, an important mediator of M1 differentiation [12].

Thus, in the present study we propose to investigate the epigenetic mechanisms HDAC-dependent that underlies the phenotypic plasticity of BMMP both in vitro and in vivo. Indeed, in vitro experiments showed that the CD11b^low^/Ly6C^low^ is the selective target for iHDAC. Upon macrophage differentiation has occurred, HDAC activity was able to modulate cell shape plasticity and function, indicating that HDAC activity is a key component of monocyte/macrophage plasticity and behavior. When topically added to skin wounds iHDAC was able to recapitulate the behavior observed in vitro and enhance wound closure and tissue repair. These results describe for the first time that the phenotypic and functional plasticity of monocytes and macrophages is, at least in part, HDAC dependent and has the potential to successfully modulate the inflammatory response in vivo. Our findings provide the biological basis that connects chromatin structure to phenotypic plasticity, which in turn, may become a tractable therapeutic strategy in further translational studies.

## Methods

### Cell culture

C57BL/6 male mice, 2–4 months old, were euthanized and the femurs were dissected, the marrow cells were obtained by flushing and seeded in α-MEM medium supplemented with 5% fetal bovine serum (FBS). The cells were kept for 1 h in a humid incubator with 5% CO_2_ at 37 °C to separate the non-adherent populations. Supernatant cells were then plated at the density of 10^6^ cells per well in α-MEM medium containing 100U/ml penicillin, 100 μg/ml streptomycin, supplemented with 5% FBS and subdivided into the following experimental groups: GM-CSF, GM-CSF + iHDAC (Trichostatin A at 10 nM). Cells were grown in 5% CO_2_ humidified incubator at 37 °C for 48 h, 72 h, 7 or 13 days. All the procedures were approved by the local ethical committee under the CEUA protocol 077/18.

### Flow cytometry analysis

For the phenotypic analysis, the supernatant cells were harvested after 24 h, 48 h, 72 h or 7 days of culture, washed twice with Phosphate Buffer Solution (PBS), pH 7.2, containing 3% FBS, quantified and their concentration adjusted to 10^6^ cells/well. We have used only the cells found in the supernatant because upon GM-CSF addition to the culture medium, the myeloid cells adhere to the plastic substrate which may lead to interferences in cell counting. To saturate Fc receptors, all cells were incubated with the Fc blocker antibody (clone 2.4G2 obtained from the Rio de Janeiro Cell Bank, Inmetro, Rio de Janeiro, Brazil) for 10 min before adding specific monoclonal antibodies (mAbs). For cell surface markers we used a mix of the following mAbs: FITC-labeled CD11b and PE-labeled LyC6 PercP-labeled Gr1 and PE-labeled CD115 (all from BD Bioscience, San Jose, CA), at 4 °C for 15 min.

For intracellular staining the cells previously stained with mAbs FITC-labeled CD11b and PE-labeled LyC6 were fixed with 2% paraformaldehyde for 20 min, permeabilized with PBS + 5% BSA (BSA: Bovine serum albumin) + 0.2% Saponin for 15 min and stained with primary polyclonal antibodies anti-H4ac (antibody anti-acetylation of histone four from Millipore) diluted in PBS + 0.2% Saponin solution for 1 h. Cells were washed twice and incubated with the secondary antibodies Alexa conjugated with fluorochrome 647 and Alexa conjugated with fluorochrome 488 (Thermo Fisher Scientific) diluted in PBS + 0.2% Saponin for 1 h. Cells were washed for further acquisition.

Cells were acquired on FACSCanto flow cytometry (BD Biosciences) and analyzed using FACSDiva software (Version 8.0) or FlowingSoftware (version 2.5.1). The gates were set as shown in Additional file 1: Figure S1.

#### Analysis of cell morphology

Using ImageJ software, the long axis and short axis of each cell were manually traced and measured. The long axis was defined as the longest length of the cell, and the short axis was defined as the length across the nucleus in a direction perpendicular to the long axis. The ratio of the two axes was determined to be the elongation factor and 200 cells per group were counted through Fiji software.

### Immunocytochemistry

After 13 days in culture, the adherents cells on coverslips were washed in PBS and fixed in 4% paraformaldehyde for 10 min at room temperature (RT), washed with PBS and permeabilized with PBS + 0.2% Triton X-100 for 5 min. The samples were washed with PBS, Fc receptor blockade was performed for 10 min with 200 μl of 24G2 cell line supernatant and the cells were washed with PBS and PBS + 5% BSA for 1 h. Cells were washed with PBS and incubated with 50 μl of the primary antibodies in PBS/BSA at 4 °C overnight. F4/80 (1:100) and H4ac (1:200) (from Millipore) antibodies were used. Upon overnight incubation the cells were washed with PBS and incubated with 50 μl of the secondary antibodies anti-goat Alexa-488 (1:800) and anti-rabbit Alexa-546 (1:800) for 2 h at RT. The nuclei were stained using DAPI. The coverslips were mounted on slides with ProLong Diamond Antifade Mountant (Thermo Fisher Scientific), analyzed by confocal microscope (LEICA TCS SP5) and the images were processed with ImageJ software.

#### Immunohistochemistry

Skin biopsies were fixed in formalin and embedded in paraffin. Four-micrometers-thick sections were deparaffinized, rehydrated and antigen retrieval in Trilogy buffer (Cell Marque). After neutralization of the endogenous peroxidase with 3% H_2_O_2_ for 15 min, the sections were incubated with protein block solution (PBS containing 10% BSA, 8% skim milk, 0.1% Triton X-100) for 1 h before undergoing incubation with the primary antibodies. The following antibodies were adopted for immunohistochemistry: goat anti-mouse arginase-1 (1:100—V-20—Santa Cruz Biotechnology), rabbit anti-mouse iNOS (1:200—N-20—Santa Cruz Biotechnology), rabbit anti-mouse H4K16ac (1:200—Cat. 17-211—Millipore). Antibodies were detected with anti-goat HRP (1:800—Thermo Fisher) and anti-rabbit HRP (1:1000—Thermo Fisher), using diaminobenzidyne as the chromogen. Sections were counterstained with Harris’ hematoxylin.

For immunofluorescence, skin biopsies were fixed in formalin, washed and after cryoprotected in 30% sucrose solution. The tissues embedded in OCT compound (Sakura) were snap frozen in liquid nitrogen and 10 micrometers thick cryostat section were washed in PBS, and incubated in blocking solution (0.3% PBS/Triton X-100 + 4% BSA) for 1 h at room temperature. Subsequently, the sections were incubated overnight with the primary antibodies rat anti-mouse F4/80 (1:200—BM8—Santa Cruz Biotechnology) and rabbit anti-mouse H4ac (1:200—cat. 06-866—Millipore). Next day, the sections were washed in PBS and incubated for 2 h in secondary antibodies: 488 Alexa-fluor goat anti-rat (1:800—Thermo Fisher) and 647 Alexa-fluor chicken anti-rabbit (1:800—Thermo Fisher). The sections were washed in PBS, counterstained with DAPI (300 nM—Sigma) and cover slipped with Prolong Gold antifade (Thermo Fisher Scientific). The images were acquired by confocal microscopy (Leica Microsystems).

### Western blot

For iNOS protein quantification, cells were maintained in culture for 18 days. For Arginase-1 protein quantification, wound skin were collected at 7 days after wounding. The protein extraction was performed with RIPA buffer plus protease inhibitor and denaturated in sample buffer. The proteins were resolved by SDS-PAGE (4% stacking and 10% running) at room temperature. Protein transfer was performed for 2 h onto a PVDF membrane. Membrane blocking was performed with 5% milk powder in 0.1% PBT at room temperature and further incubated with primary antibodie anti-iNOS 1:200 and anti-tubulin 1:2000 at 4 °C overnight. Membranes were washed with 0.1% PBT, incubated with the HRP-conjugated secondary antibodies (anti-goat, anti-rabbit and anti-mouse) for two hours at room temperature under stirring and then washed again three times with 0.1% PBT. Reaction development was monitored using ImageQuant LAS 500 (G.E. Healthcare) using SuperSignal developer West Pico Chemiluminescent Substrate (Thermo Scientific). The images were processed in ImageJ software and analyzed in GraphPad Prism 5.0 software.

### Statistical analysis

Statistical analysis were performed using GraphPad Prism 5.0 software and using Student’s T test, One-way ANOVA or Two-way ANOVA with Bonferroni correction depending on the experiment. Differences were considered statistically significant at *P < 0.05, **P < 0.01, ***P < 0.001. The results are presented as the means ± SD.

## Results

### HDAC activity blockade leads to the expansion of the bone marrow myeloid CD11b^low^/Ly6C^low^ subset for up to 7 days in culture

In our previous work we have demonstrated, for the first time, that the inhibition of HDAC activity shifts the myeloid differentiation dynamics leading to the expansion of the CD11b^low^/Gr1^low^ subset [12]. Upon 15 days in culture, we observed the presence of very elongated macrophages harboring a mixed M1/M2 phenotype. In the present work we have used the surface marker Ly6C to better understand the relevance of HDAC activity to the generation of classic/inflammatory CD11b^+^/Ly6C^high^ or non-classical/resolving CD11b^+^/Ly6C^low^ monocyte subsets [13] in the absence of any pro or anti-inflammatory stimuli. We hypothesized that HDAC activity blockade would favor the expansion of Ly6C^low^ progenitors that would give rise to the very elongated macrophages upon 7 days in culture. For this, non-adherent bone marrow cells were cultured in the presence of GM-CSF to induce myeloid differentiation of the hematopoietic progenitors. In addition, the cells were exposed to 10 nM TSA (iHDAC) and cultured up to 7 days. Firstly, we used the surface markers Gr1 (recognizes both Ly6G, granulocytes, and Ly6C, granulocytes and monocytes) and CD115 (M-CSF receptor and identifies monocytes and macrophages) to better understand whether HDAC activity is necessary for monocytic or granulocytic differentiation. Upon 72 h in culture, we observed that HDAC activity blockade led to an increase in percentage of cells in the P15 regions (Gr1^low^/CD115^−^), P21 (Gr1^low^/CD115^low^), and P23 (Gr1^low^/CD115^high^) when compared to the GM-CSF group (Additional file 1: Figure S1). These results demonstrate that iHDAC target population is not the population that display higher levels Gr1.

**Fig. 1:**
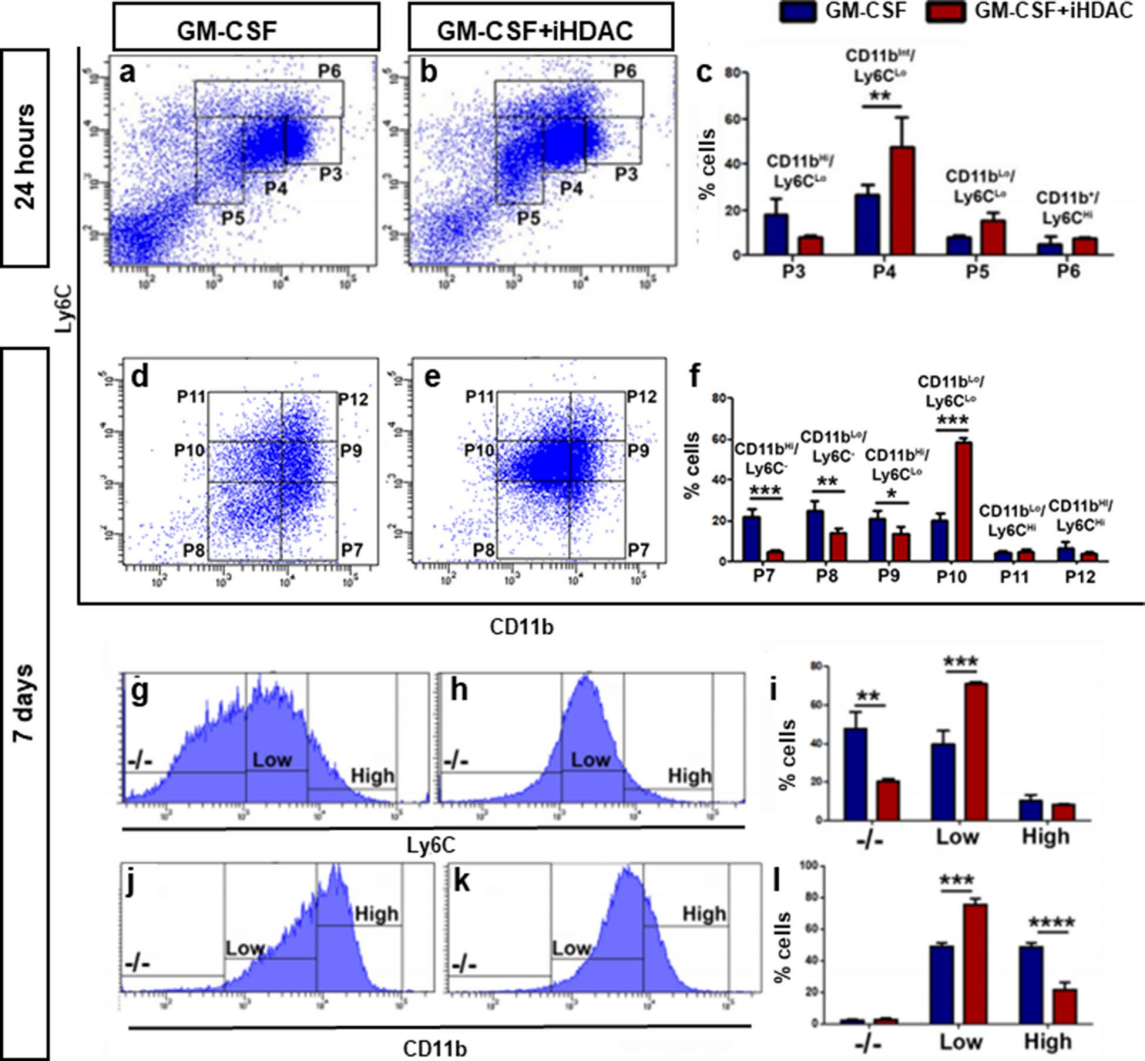
HDAC activity is necessary for myeloid differentiation. Non-adherent bone marrow cells were treated with GM-CSF (10 ng/ml) and/or iHDAC (10 nM TSA) and analyzed after 24, 72 and 7 days of culture. **b** Region P2 for exclusion of dead cells and cell debris. Phenotypic analysis of hematopoietic cells by two classical markers of the myeloid lineage, CD11b and Ly6C, at 24 h (**a**–**c**) and 7 days (**d**–**f**). Histogram of Ly6C (**g**–**i**) and CD11b (**j**–**l**) subsets upon 7 days in culture. n = 3 animals per group; Data are mean ± SD. Statistically significant differences p < 0.05, **p < 0.01, ***p < 0.001, ****p < 0.001 by the two-way repeated measures ANOVA followed by the test of Bonferroni to correct the value of p

Therefore, we ruled out the hypothesis that activity HDAC is required for granulocyte differentiation and hypothesized that the main target for iHDAC would be the monocytic population. Because myeloid progenitors, monocytes and macrophages display different levels of the surface markers CD11b and Ly6C down the differentiation pathway, we used these two markers to track BMMP differentiation in the absence of HDAC activity. Upon 24 h in culture, GM-CSF promoted the differentiation of cells of the myeloid lineage of CD11b^+^ and Ly6C^+^ (Fig. 1a) and iHDAC did not prevent the myeloid differentiation induced by GM-CSF. Interestingly, iHDAC in the presence of GM-CSF promoted an increase in the population in the P4 region (CD11b^int^/Ly6C^low^; Fig. 1b, c). In the GM-CSF group, however, we found the cells homogeneously distributed between the P4 (CD11b^int^/Ly6C^low^) and P3 (CD11b^high^/Ly6C^low^) regions (Fig. 1a, c). After 7 days of culture, we observed that while GM-CSF treated myeloid cells were homogeneously distributed among the P4 (CD11b^high^/Ly6C^−^), P5 (CD11b^low^/Ly6C^−^), P6 (CD11b^high^/Ly6C^low^) and P7 (CD11b^low^/Ly6C^low^) (Fig. 1d, f) regions, iHDAC treated cells were mostly found in the P10 region (CD11b^low^/Ly6C^low^) (Fig. 1e, f). In fact, the fluorescence intensity of the CD11b and Ly6C markers in the iHDAC group was low along the 7 days of analysis when compared to GM-CSF alone (Fig. 1g–l). These results show that HDAC activity is required for myeloid differentiation, and in synergy with GM-CSF, drives the differentiation of inflammatory monocytes. The iHDAC, in contrast, favors the expansion of myeloid progenitor of the CD11b^low^/Ly6C^low^ subset cells for longer in culture.

### HDAC activity blockade targets bone marrow myeloid precursors of the CD11b^low^/Ly6C^low^ subset and leads to histone H4 hyperacetylation

**Fig. 2:**
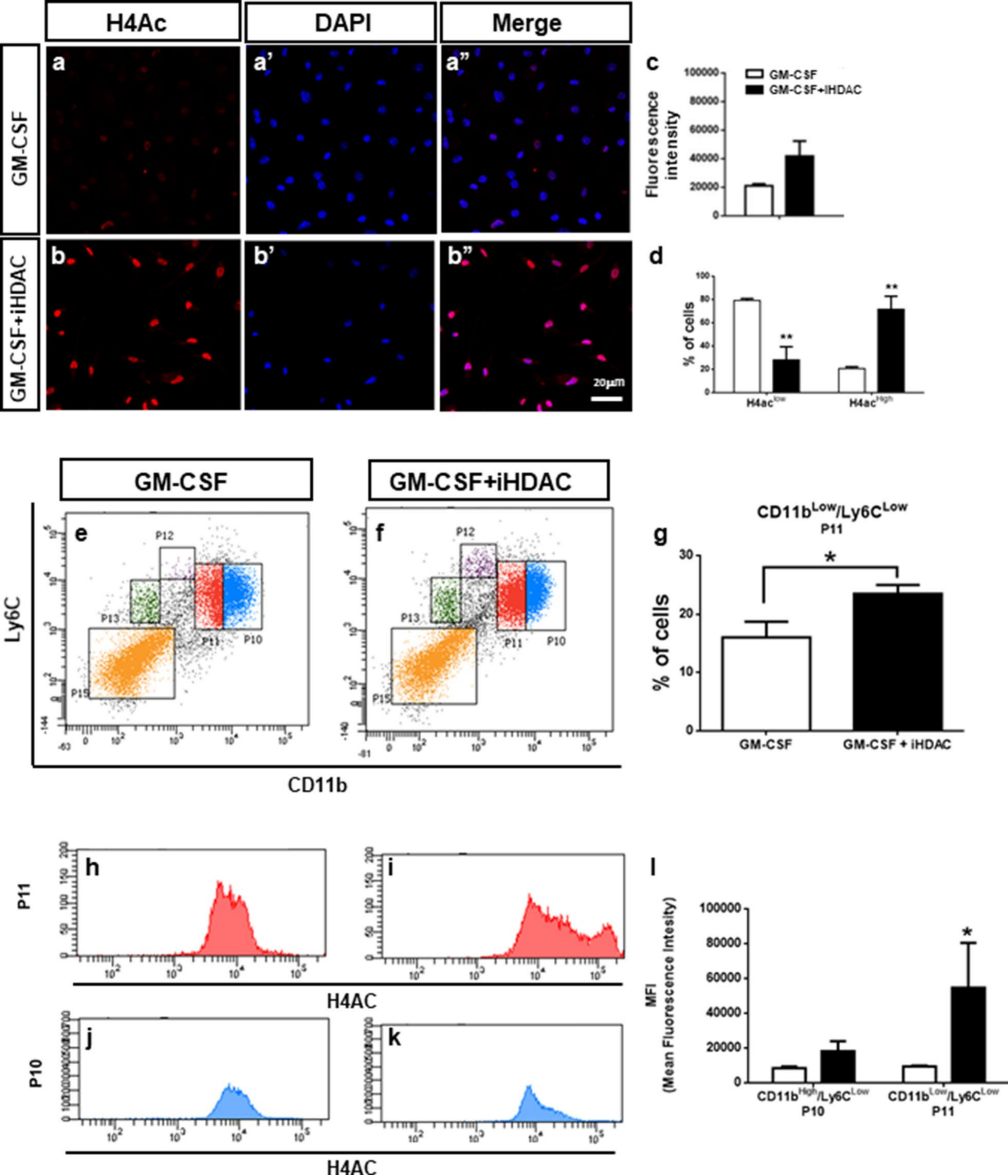
The CD11b^low^/Ly6C^low^ subset is the specific target of iHDAC. Analysis after 48 h of culture by flow cytometry of non-adherent bone marrow cells from GM-CSF or GM-CSF + iHDAC treated groups. **a**, **b** Immuno positive cells for acetylated histone H4 were observed only in the GM-CSF + iHDAC treated cells (**b**; **b**’ DAPI; **b**” merge). GM-CSF treated cells did not stain for acetylated histone H4 (**a**; **a**’ DAPI; **a**” merge). **c**, **d** Acetylated levels of histone H4 were significantly higher in iHDAC treated cells. Phenotypic analysis of myeloid cells through the CD11b and Ly6C showed that the Cd11b^low^/Ly6C^low^ harbor significant higher levels of acetylated histone 4 (**e**–**g**). Representative MFI histogram of P10 (CD11b^high^/Ly6C^low^) and P11 (CD11b^low^/Ly6C^low^) subsets shows significant increase of acetylated histone H4 levels in the P11 subset (**h**–**l**). n = 4 animals per group; Data are mean ± SD. Differences statistically significant, *p < 0.05, **p < 0.01, by the two-way ANOVA followed by the Bonferroni test for correction of the p value

We then asked which bone marrow myeloid subset would be the target of HDAC activity. For this BMMP were cultured in the presence of GM-CSF and/or iHDAC for 48 h and chromatin acetylation levels were analyzed by immunostaining. In fact, cells exposed to iHDAC were immuno positive for acetylated histone H4 (H4Ac) when compared to the control group (Fig. 2a–d). To characterize which subpopulation of BMMP would be the specific target for iHDAC, we analyzed the levels of H4Ac along with the surface markers CD11b and Ly6C after 48 h in culture. In fact, it was possible to observe that H4Ac levels were increased in the CD11b^low^/Ly6C^low^ subset only in the iHDAC treated group (Fig. 2e–g). In accordance, MFI showed that CD11b^low^/Ly6C^low^ subset displayed higher levels of acetylated H4 histone in the GM-CSF + iHDAC group (Fig. 2h, i, l). In contrast, the acetylation levels of H4 histone were not different in the CD11b^high^/Ly6C^low^ subset between control and iHDAC treated groups (Fig. 2j, k, l). These data show that of all bone marrow subpopulations, the CD11b^low^/Ly6C^low^ subset is the specific target of iHDAC, which in turn, leads to chromatin changes due to histone H4 hyperacetylation.

### iHDAC treated macrophages may become pro-inflammatory iNOS producing cells whether cell shape shortening is evoked

**Fig. 3:**
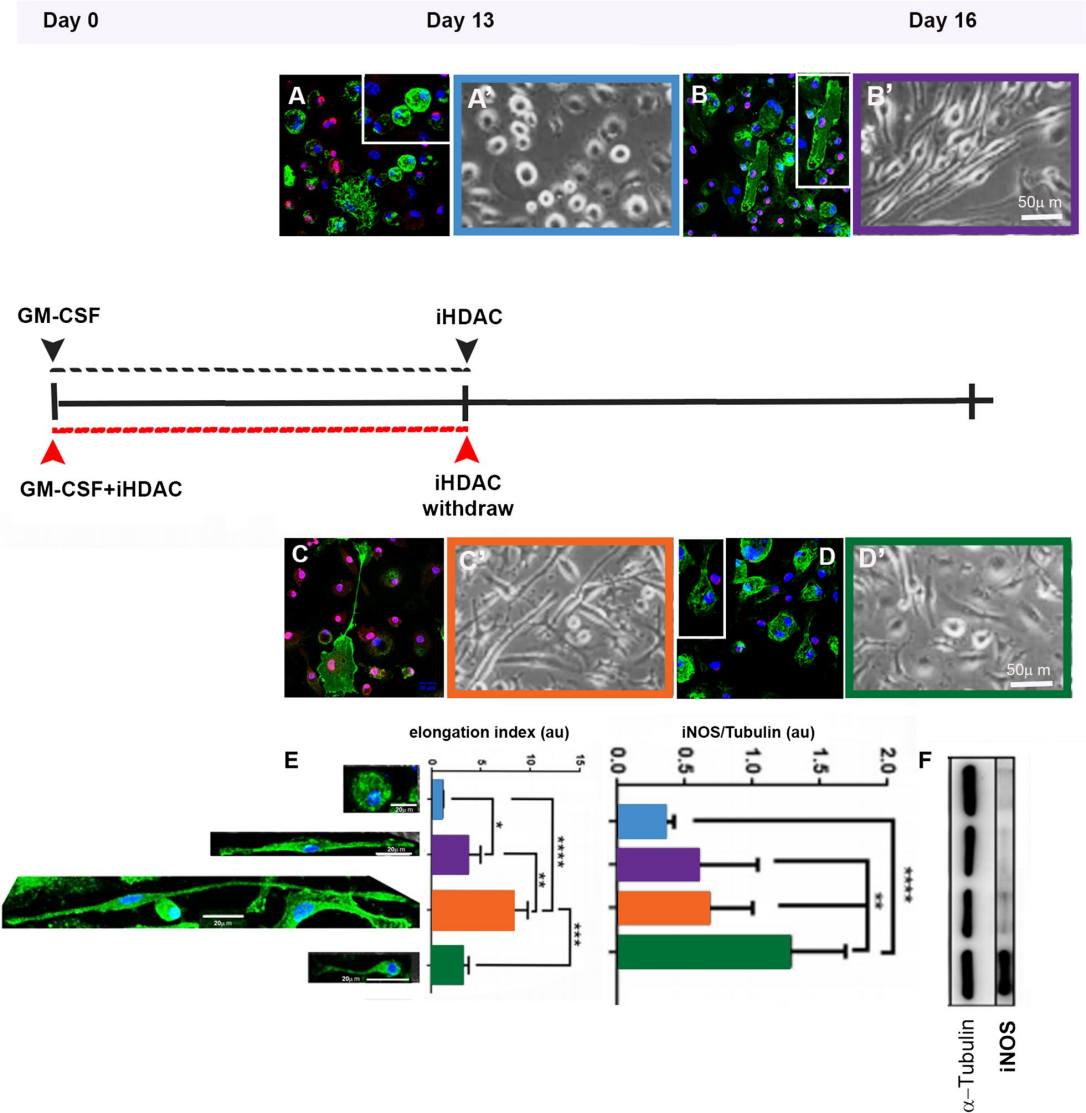
HDAC activity modulates macrophage cell shape and function. BMMP cells were grown in the presence of GM-CSF (**A**’) or GM-CSF + iHDAC (**c**’) for 13 days. At day 13 the iHDAC was added (**B**’) to or removed (**D**’) from the culture medium. Macrophages were positive for F4/80 (green) and H4Ac (red) (**B**, **C**) and were positive only for F4/80 (green) (**A**, **D**); blue: DAPI. The elongation index was calculated to every condition (**E**) and compared to the levels of iNOS protein by western blot (**F**). n = 4 animals per group; Data are mean ± SD. Statistically significant differences, *p < 0.05, **p < 0.01, ***p < 0.001, ****p < 0.0001 by repeated two-way ANOVA followed by Bonferroni test for correction of p value

In our last work we demonstrated that the blockade of HDAC activity during 15 consecutive days led to the generation of elongated macrophages with mixed phenotype M1/M2 [12]. Then, we hypothesize that HDAC activity would be a key player for the maintenance of cellular plasticity over the time. In fact, regardless the literature has successfully characterized the existence of M1 (pro-inflammatory) and M2 (repairing/resolving) macrophages, it is still not clear whether these functional subsets arise from the same progenitor cell or from different ones. Thus, we hypothesized that HDAC activity could be, at least in part, a key player to proper confer plasticity to myeloid progenitors committed to macrophage differentiation. If in fact, if this hypothesis is corroborated, the use of iHDACs to modulate the behavior and function of macrophages in vivo might become an important strategy for tissue repair in translational studies. To test this hypothesis, we cultured BMMP under two different conditions: in the first BMMP cells were cultured in the presence of GM-CSF alone for 13 days (Fig. 3A’). At this point the macrophages presented a rounded morphology and were H4Ac negative (Fig. 3A). After this time, iHDAC was added to the medium and cells were analyzed 3 days later (Fig. 3B’ day 16). At this point, the macrophages started displaying an elongated morphology that was correlated to H4Ac positive staining (Fig. 3B).

In the second condition, BMMP cells were cultured in the presence of GM-CSF + iHDAC for 13 days (Fig. 3C’). At this point, macrophages displayed a very elongated morphology and were positive for H4Ac (Fig. 3C). After this time, iHDAC was removed from the culture medium and cells were analyzed 3 days later (Fig. 3D’ day 16). At this point, macrophages lost their elongated morphology as well as the H4Ac staining (Fig. 3D). Cells grown in the presence of GM-CSF alone for 13 days presented rounded morphology (Fig. 3A, A’) and after iHDAC addition to the culture medium, they presented a higher elongation index (Fig. 3E, purple bar) but maintained the same levels of iNOS observed in cells grown in the presence of GM-CSF alone (Fig. 3F, purple bar). However, BMMP grown in the presence of GM-CSF + iHDAC for 13 days showed an extremely elongated morphology (Fig. 3C, C’; E, orange bar) but maintained the same levels of iNOS when compared to cells grown only in the presence of GM-CSF (Fig. 3E, blue bar) or GM-CSF plus iHDAC (Fig. 3E, purple bar). In clear contrast, BMMP cultured in the presence of GM-CSF + iHDAC for 13 days and in the presence of GM-CSF alone for further 3 days, presented lower elongation index, when compared to cells grown in the presence of iHDAC for 13 days, due to a shortening in cell length (Fig. 3E, green bar). More interestingly, these cells up regulated the levels of iNOS after iHDAC was removed from the culture medium (Fig. 3F, green bar). This set of experiments shed light on two very important aspects of myeloid cells development and plasticity: the first one is that we can dissect the HDAC activity necessity along the time. At the beginning of BMMP differentiation, HDAC activity is necessary for BMMP progenitor differentiation towards pro-inflammatory subsets (Cd11b^+^/Ly6C^high^) and iHDAC leads to the expansion of CD11b^low^/Ly6C^low^. The second information is that HDAC activity is also necessary for macrophage plasticity and behavior upon differentiation has occurred. In fact, the elongated morphology of iHDAC-treated derived macrophages was tight correlated to macrophage function because more elongated macrophages express as much iNOS as GM-CSF-induced macrophages. However, iHDAC treated macrophages may become pro-inflammatory iNOS producing cells whether cell shape shortening was evoked. So, we hypothesized that this amazing property would connect cell shape control to function and plasticity, turning the HDAC activity into a putative target for tissue repair translational studies.

### HDAC activity blockade modulates bone marrow myeloid progenitors behavior in vivo upon acute injury and improves wound healing

**Fig. 4:**
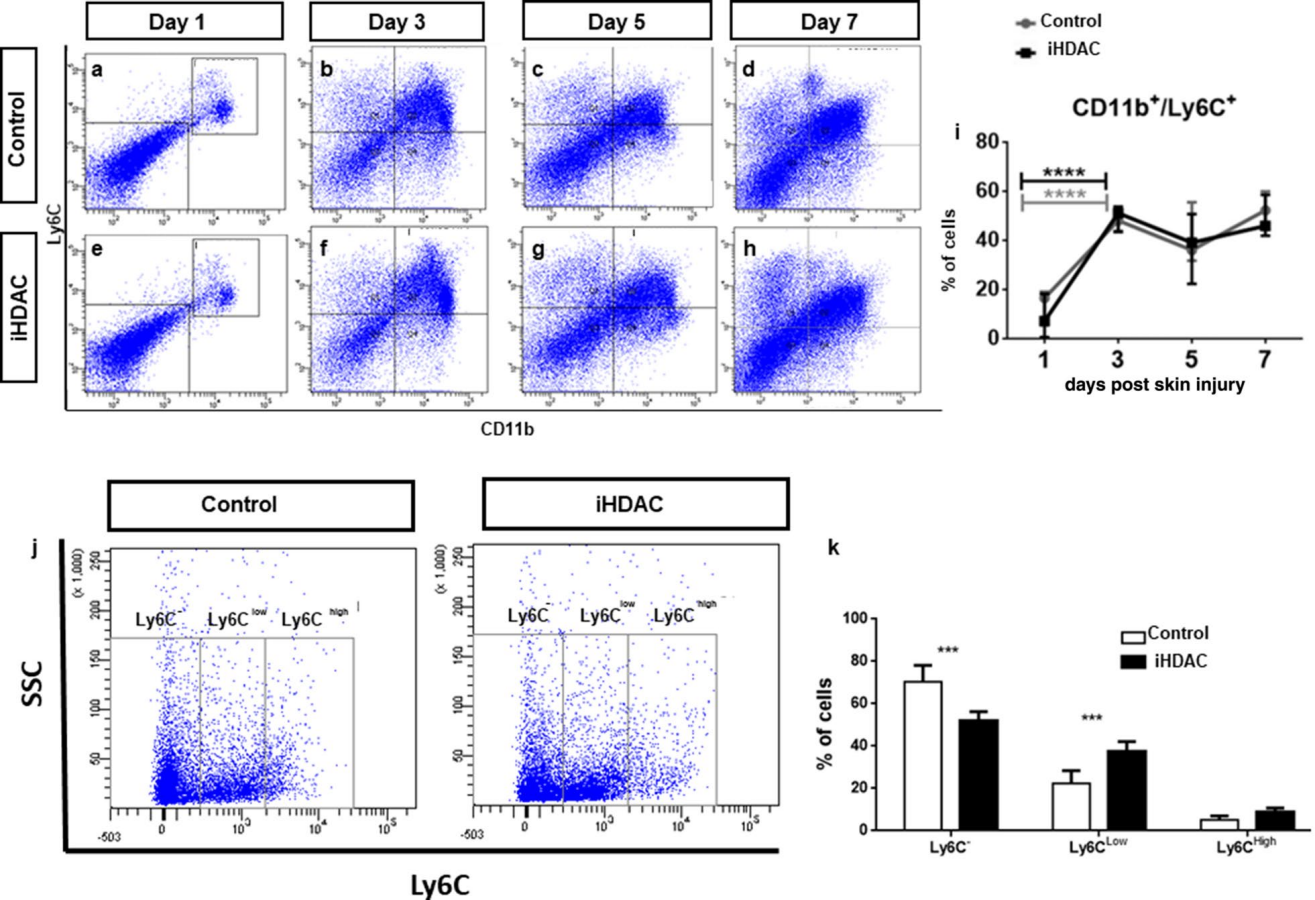
iHDAC leads to the expansion of the CD11b^low^/Ly6C^low^ subset upon wound injury. Upon skin injury the inflammatory infiltrate was quantified by flow cytometry. Although the injury mobilized peripheral blood cells between day 1 and day 3 after wound induction to compose the inflammatory infiltrate (**a**–**h**), the blockade of HDAC activity did not alter the recruitment dynamics of blood subsets observed in the control group along seven days post injury (**i**). N = 6 ***p < 0.001. However, when this population was phenotyped for the Ly6C^low^ and Ly6C^high^ subsets, we were able to observe a significant increase in the Ly6C^low^ subset in iHDAC treated skins at 7 days after injury (**j**, **k**). N = 4; ***p < 0.001, by the two-way ANOVA test followed by the test of Bonferroni to correct the value of p

**Fig. 5:**
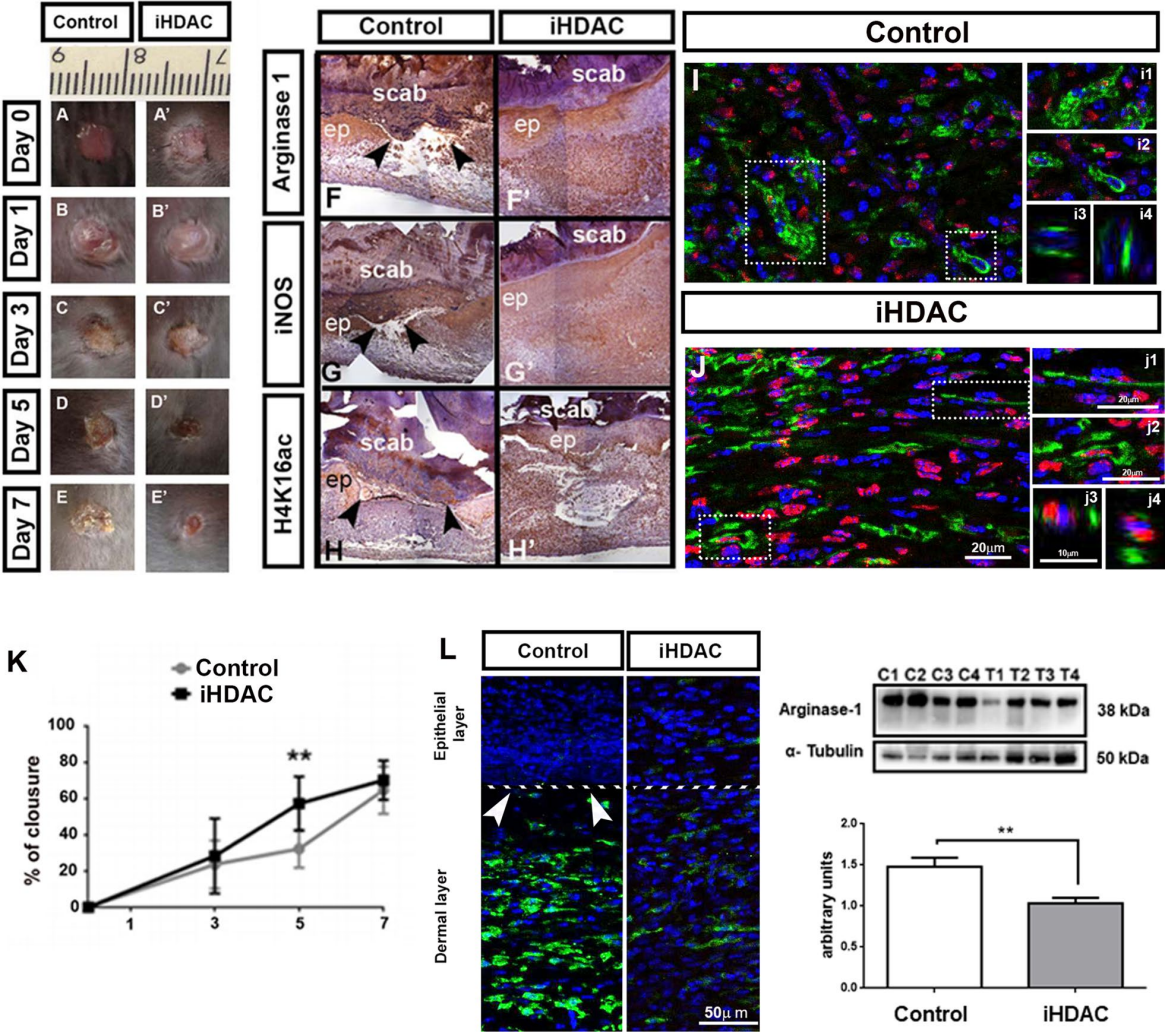
iHDAC modulates bone marrow myeloid progenitors behavior in vivo and improves wound healing. Inhibition of HDAC activity improves wound healing after 5 days of skin lesion (**A**–**E**, **K**). n = 12 animals per group. Data representative of 3 independent experiments. Data are mean ± SD. Statistically significant differences, **p < 0.01, by the two-way ANOVA test followed by the test of Bonferroni to correct the value of p. Both control and iHDAC wounds were positive for Arginase-1 (**F**, **F**’), iNOS (**G**, **G**’) and H4K16ac (**H**, **H**’). Confocal microscopy analysis revealed the presence of very elongated F4/80/H4ac positive cells only when wounds were topically treated with iHDAC (**J**, inset). Confocal analysis of optical cross sections (**j2**) in addition to XZ (**j3**) and YZ (**j4**) projections highlights that F4/80 + cells (green, **j2**) and also positive for H4Ac (red, **j2**). In contrast, confocal analysis of optical cross sections (**i2**) in addition to XZ (**i3**) and YZ (**i4**) projections highlights that macrophages in the control skin were positive only for F4/80. **K** Wound healing dynamics during 7 days upon skin injury in control and iHDAC treated wounds. **L** Immunohistochemistry of control and iHDAC treated wounds showing an enrichment of Arginase-1 + cells in control wounds in the dermal layer if compared to iHDAC treated wounds (white arrows, discontinuous epidermal/dermal layer boundary). Western blot quantification analysis showed that iHDAC treated wounds (triplicates T1, T2, T3) presented significant lower levels of Arginase-1 when compared to control group (triplicates C1, C2, C3). N = 4; **p < 0.01, by Test T

In accordance to our data, we next speculated whether iHDAC would modulate BMMP in vivo upon acute skin injury. The relevance of monocyte subsets dynamics into the skin wound as well as the relevance of macrophage role during wound healing are very well established. For this reason, we decided to test whether iHDAC topic delivery on skin wounds would recapitulate the BMMP behavior observed in vitro. If so, iHDAC might favor the expansion of CD11b^low^/Ly6C^low^ subset and also modulate macrophage cells shape towards the elongated morphology in vivo, and as consequence, improve wound healing. To assess whether inhibition of HDAC activity could modulate the phenotypic and morphological plasticity of BMMP in vivo, we monitored monocyte subsets dynamics from day 1 to day 7 post wound lesion. Upon wound induction, iHDAC was topically added to wounds once a day during 7 consecutive days and monocytes subsets dynamics was monitored at days 1, 3, 5 and 7 post lesion by flow cytometry. In fact, we did not detect any consistent difference in monocytes subsets from bone marrow, peripheral blood and wound infiltration between control and iHDAC along the time (Additional file 2: Figure S2; Additional file 3: Figure S3; Additional file 4: Figure S4, respectively). We conclude that it is unlike that topic treatment with iHDAC would be modulating the recruitment of central or peripheral monocytes into the wound and that iHDAC is good candidate for topic use as its effects were restricted to the area of delivery. For this reason, we went to characterize the BMMP subsets in the wound. Regardless we have failed to observe a significant difference in the recruitment of the CD11b^+^/Ly6C^+^ subset from peripheral blood to the wound, between control and iHDAC group (Additional file 5: Figure S5), we noticed that the CD11b^+^/Ly6C^low^ subset was significantly increased in the iHDAC treated wounds (Fig. 4). Then, we asked whether such enhancement in the CD11b^+^/Ly6C^low^ subset into the wounds would correlate with an improvement in the wound closure. For this we monitored the wound healing dynamics up to 7 days. In fact, after 5 days of topic treatment, the iHDAC group presented a higher percentage of healing when compared to the group of animals treated with solvent solution (Fig. 5A–E, K).

In accordance, histopathological sections of control and iHDAC treated wounds uncovered remarkable qualitative and quantitative differences in cutaneous lesions. While control and iHDAC treated wounds were positive for iNOS, Arginase-1 and H4ac (Fig. 5A–F), we observed the presence of very elongated F4/80^+^/H4Ac^+^ macrophages only in iHDAC treated wounds (Fig. 5J, J1–J4) that correlated with significant decrease in Arginase-1 + cells in the dermal layer and lower levels of Arginase-1 (Fig. 5L). It is also possible to note that iHDAC treated wounds presented full reconstitution of both epithelial and dermal layers (Fig. 5L) while control wounds still presented a discontinuation between both layers (Fig. 5L, white head arrows). Regardless macrophages from control wounds were positive for F4/80, they did not presented an elongated morphology neither were positive for H4Ac (Fig. 5I, I2–I4). These results clearly show that topic iHDAC treatment recapitulated the effect generated in vitro on BMMP subsets dynamics as well as on macrophage cell shape. We conclude that the topic treatment with iHDAC recapitulated the effects observed in vitro leading to macrophage cell shape changes and promoting the expansion of the Ly6C^low^ subset in vivo.

## Discussion

HDACs were first described in yeast [14–16] and since their catalytic activity was first described [14], many pharmacological inhibitors (iHDACs) have been identified and tested in vitro and in vivo [17]. By presenting an ubiquitous and redundant expression pattern, HDACs have received much attention as putative therapeutic targets for several clinical approaches, including inflammation [18]. However, although iHDACs are widely described as potent anti-inflammatory drugs, it is still unclear how their action modulates the complex interplay among different players during the inflammatory response.

In this sense, we hypothesized that the blockade of HDAC activity may lead to the maintenance of a given dynamic chromatin state that confer more plasticity to the macrophages that will be differentiated from BMMP, opening an interesting avenue of research on the potential of iHDACs as a candidate to reverse the deleterious effects of a persistent inflammation such as found in metabolic disorder and skin injuries. Many studies have shown that the imbalance in the modulation of inflammation by cells of the innate immune system plays critical roles in metabolic syndromes and responds to the deleterious effects generated in the medium and long term as consequence of chronic inflammation [19, 20]. Another study demonstrated that the addition of TSA to bone marrow cells favored the increase of myeloid lineage defined as cKit + CD11b + Gr + Lyc6 + in the presence of GM-CSF in detriment of differentiation, both in vitro and in vivo [21]. Our results are in accordance with the authors concerning the expansion of the myeloid progenitors and enhanced acetylation of histone in bone marrow cells in presence of GM-CSF and TSA. Considering that GM-CSF is a multipotent hematopoietic cytokine that targets very early myeloid progenitors, we demonstrated for the first time that the CD11b^low^Ly6C ^low^ is the responsive subset of progenitors displaying putative anti-inflammatory characteristics.

Following this line of reasoning, we have demonstrated in a previous work that the blockade of HDAC activity in BMMP led to the generation of plastic macrophages displaying a mixed phenotype M1/M2 [12]. In this study it was clear that the establishment of a HDAC activity-dependent epigenetic landscape during BMMP differentiation is crucial for the functional programming of proinflammatory macrophages, and the blockade of HDAC activity leads to the generation of functionally more plastic macrophages. This perspective opens important avenues of research since the modulation of HDAC activity appears as a putative therapeutic target for the modulation of the inflammatory response in vivo. In fact, to use the pharmacological knockdown of the enzymatic activity of HDACs is a very tractable strategy because allows us to translate our findings to scenarios where the inflammatory response would be faced as a challenge to our model. Indeed, we consistently showed that HDAC activity is key component of BMMP and macrophage plasticity. If used early during the differentiation pathway cascade, iHDAC may successfully amplify early myeloid progenitors that will be further committed to the monocyte lineage. If used later, upon macrophage differentiation took place, iHDAC may contribute to modulate cell shape and function, conferring remarkable plasticity to macrophages proper respond to environmental cues. We highlight that the phenotype obtained upon iHDAC treatment was independent on the presence of pro-inflammatory molecules (M1), such as LPS, or anti-inflammatory (M2) molecules such as IL-4. It is therefore a mechanism that bridges phenotypic plasticity with functional plasticity through the modulation of HDAC activity. Our findings are in agreement with results previously published that showed the remarkable phenotypic plasticity associated to macrophage cell shape [22].

Such kind of mechanism would be of great clinical interest because has the potential to modulate BMMP in vivo and drive the inflammatory response towards the resolution. In fact, the skin wound healing is a proper scenario to test the efficiency of iHDACs in modulating the behavior of less differentiated BMMPs from the CD11b^low^/Ly6C^low^ subset. We chose this scenario because monocyte dynamics upon wound injury is very well understood. In fact, mouse monocytes from the Ly6C^high^ subset are efficiently recruited upon injury and give rise to inflammatory macrophages [4, 6]. In contrast, Ly6C^low^ monocytes patrol blood vessels and are key players during early response to injuries [7, 8]. Different studies have shown that the Ly6C^high^ subset can convert into the Ly6C^low^ subset [23, 24] and upon tissue injury these monocytes can infiltrate into the damaged tissue where they will exert different physiological functions. While the Ly6C^high^ subset will secrete pro-inflammatory mediators [25], the Ly6C^low^ subset will secret a range of pro healing mediators [26] and its early recruitment to the injury site may lead to an enhancement of tissue remodeling and repair [27]. However, it is still unknown how these two different subsets of monocytes contribute to tight defined macrophage phenotypes. In this work we propose that the CD11b^low^/Ly6C^low^ subset is a selective target for iHDAC. We propose this hypothesis because we noticed the increase in the CD11b^low^/Ly6C^low^ subset if wounds were topically treated with iHDAC. Because we also did not notice any significative difference in bone marrow mobilization and peripheral blood recruitment of monocytes, we conclude that upon infiltration, the blockade of HDAC activity promoted the expansion of the CD11b^low^/Ly6C^low^ subset exclusively in the iHDAC treated wounds.

Our results also add a new layer of information on the papers published by Spallotta 2013 [28, 29]. The authors showed that TSA enhanced skin repair due to a positive effect on keratinocytes proliferation without paying attention to the role of BMMP cells. In addition to this effect on wound healing, our work sheds light on the key role of iHDAC on BMMP cells and indicates a keratinocyte independent effect of iHDAC as the modulation of Arginase-1 cells took place at the dermal layer instead of epithelial layers where keratinocytes are found. In fact, these findings are very interesting because they open the perspective of therapeutic use of iHDACs in conditions where the inflammatory response must to be tightly balanced. For example, successful approaches have been performed in order to enhance the recruitment of Ly6C^low^ subset to promote tissue repair and regeneration [27, 30, 31]. In this sense, our work is the first one to propose a strategy to modulate monocyte behavior by epigenetic mechanisms HDAC-dependent in vivo. Future studies should therefore be directed to the approach of the therapeutic administration of iHDACs in order to modulate monocyte cell behavior to favor the expansion of the Ly6C^low^ subset to favor tissue remodeling and regeneration. Our work, therefore, provides the biological bases that connect the chromatin structure and the phenotypic plasticity, which in turn, may become an important clinical strategy in translational studies.

## Conclusions

Our work describes for the first time in the literature the relevance of HDAC activity for bone marrow myeloid progenitors behavior and plasticity. We conclude that HDAC is required early during myeloid development to the proper differentiation of pro inflammatory Ly6C^high^ monocytes. In the absence of HDAC activity, however, the Ly6C^low^ subset is expanded. Upon differentiation, HDAC activity is a key modulator of cell shape and functional plasticity as the shortening of macrophage length upon iHDAC withdraw up regulated the levels of iNOS, clearly connecting macrophage cell shape and function. In addition, we conclude that topically delivered iHDAC recapitulated the monocyte and macrophage behavior observed in vitro. Remarkable this effect was straight correlated to the presence of CD11b^low^/Ly6C^low^, very elongated F4/80mpositive macrophages and wound closure enhancement.

## Additional files

**Additional file 1: Figure S1.** Gating strategy for flow cytometry analysis. In this sample gating, (A) cells were first gated for singlets (FSC-H vs. FSC-A) (B) and myeloid cells (SSC-A vs. FSC-A). (C) The unstained control was used to determine negative cell population (D and E) and the fluorescence minus one (FMO) control was used to identify and gate cell populations.

**Additional file 2: Figure S2.** Inhibition of HDAC activity increases the population of monocyte progenitors (Gr1^low^/CD115^−^), monocytes (Gr1^low^/CD115^low^) and macrophages (Gr1^low^/CD115^hi^). (A) and (B) Phenotypic analysis of hematopoietic cells by two classical markers of myeloid lineage, CD115 and Gr-1, after 72 h of culture. (C) Percent of cells in the regions (P) delimited in the dot plots. n = 3 animals per group; The data are the mean ± SD. Statistically significant differences, *p < 0.05, **p < 0.01, ***p < 0.001, by the ANOVA test of two-way repeated measures followed by the Bonferroni test for *p* value correction.

**Additional file 3: Figure S3.** Topic iHDAC on wounds does not mobilize bone marrow. Upon wound induction bone marrow dynamics was monitored by flow cytometry for 7 (A–H) days and any significant difference was detected in bone marrow subsets dynamics (I–L). N = 6.

**Additional file 4: Figure S4.** Topic iHDAC on wounds does not mobilize peripheral blood cells. Upon wound induction blood cells subsets were monitored by flow cytometry for 7 days (A–H) and any significant differences were detected in different blood subsets (I, J). N = 6.

**Additional file 5: Figure S5.** iHDAC does not alter peripheral blood subsets dynamics in wounds upon infiltration along the time. Upon wound induction, different subsets from peripheral blood infiltrated into the wounds were monitored for 5 days (A–F). Any significant difference was detected between control and iHDAC wounds with regard the infiltrating cells along the time (G–I). N = 6.

## Data Availability

All data generated or analyzed during this study are included in this published article and its Additional files.

## Abbreviations

BMMP: bone marrow myeloid progenitors
HDAC: histone deacetylase
iHDAC: HDAC inhibitor
HSC: hematopoietic stem cell
CMPs: common myeloid progenitors cells
GMP: granulocyte–macrophage progenitors
MDP: monocyte-dendritic progenitors
cDC: classical dendritic cells
GM-CSF: granulocyte and macrophage colony stimulating factor
MNp: specific monocytes progenitors
FBS: fetal bovine serum
H4Ac: acetylated histone H4
iNOS: inducible nitric oxide synthase
TSA: trichostatin A
LPS: lipopolysaccharide
Il-4: interleukin 4
