# Supplementary figures and images for: The epigenome as a putative target for skin repair: the HDAC inhibitor Trichostatin A modulates myeloid progenitor plasticity and behavior and improves wound healing
Source: J Transl Med. 2019 Jul 31;17:247. doi: 10.1186/s12967-019-1998-9 (PMC6668089; doi:10.1186/s12967-019-1998-9)

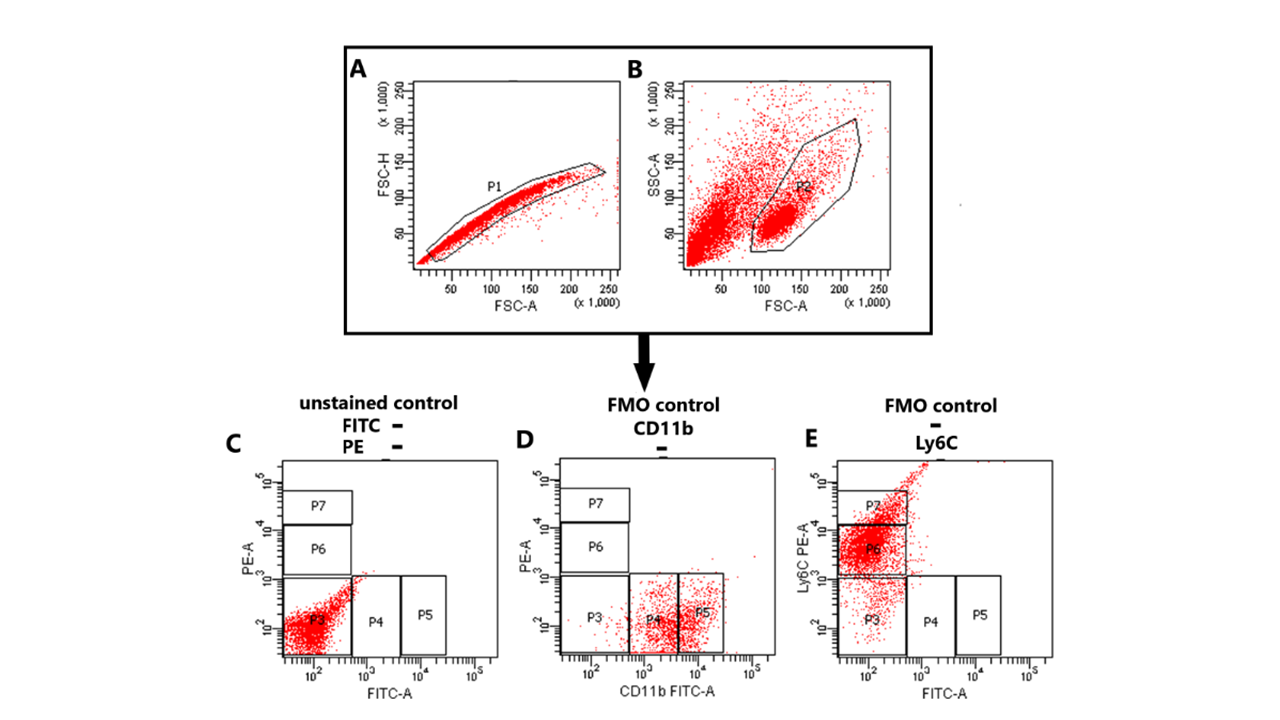

Supplement: Supplementary file 1 — Additional file 1: Figure S1. Gating strategy for flow cytometry analysis. In this sample gating, (A) cells were first gated for singlets (FSC-H vs. FSC-A) (B) and myeloid cells (SSC-A vs. FSC-A). (C) The unstained control was used to determine negative cell population (D and E) and the fluorescence minus one (FMO) control was used to identify and gate cell populations. [file 12967_2019_1998_MOESM1_ESM.tif]

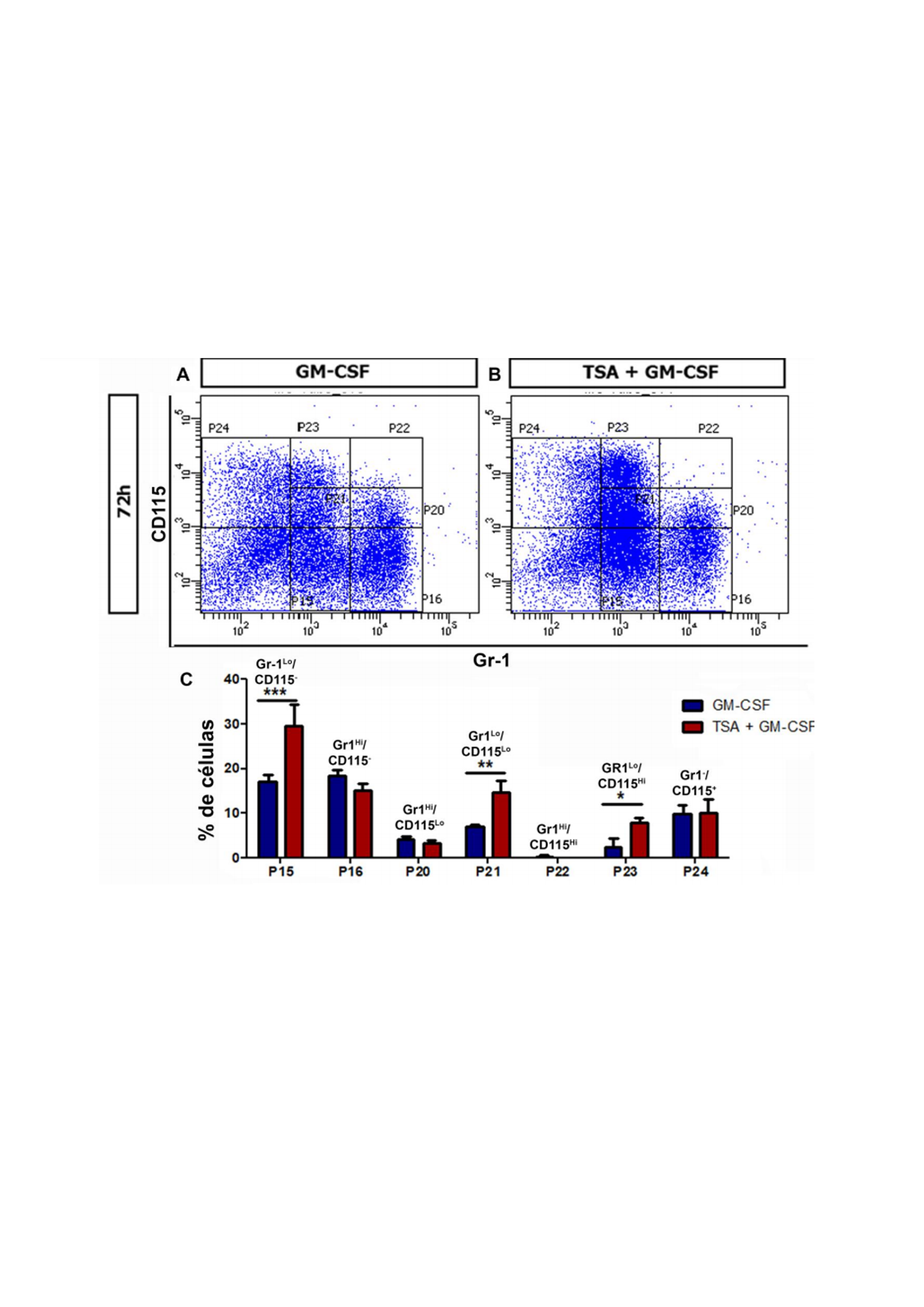

Supplement: Supplementary file 2 — Additional file 2: Figure S2. Inhibition of HDAC activity increases the population of monocyte progenitors (Gr1low/CD115−), monocytes (Gr1low/CD115low) and macrophages (Gr1low/CD115hi). (A) and (B) Phenotypic analysis of hematopoietic cells by two classical markers of myeloid lineage, CD115 and Gr-1, after 72 h of culture. (C) Percent of cells in the regions (P) delimited in the dot plots. n = 3 animals per group; The data are the mean ± SD. Statistically significant differences, *p < 0.05, **p < 0.01, ***p < 0.001, by the ANOVA test of two-way repeated measures followed by the Bonferroni test for p value correction. [file 12967_2019_1998_MOESM2_ESM.tif]

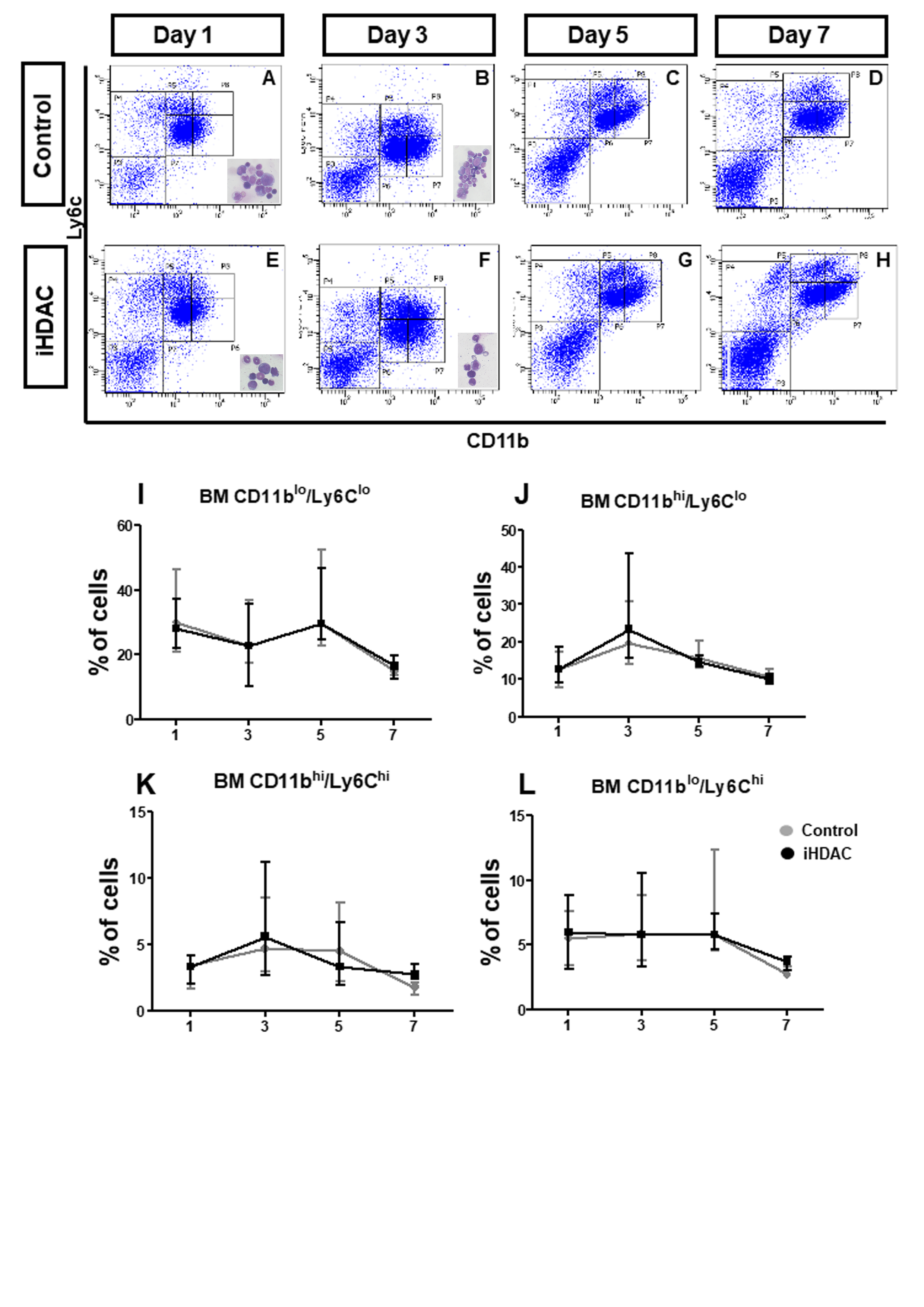

Supplement: Supplementary file 3 — Additional file 3: Figure S3. Topic iHDAC on wounds does not mobilize bone marrow. Upon wound induction bone marrow dynamics was monitored by flow cytometry for 7 (A–H) days and any significant difference was detected in bone marrow subsets dynamics (I–L). N = 6. [file 12967_2019_1998_MOESM3_ESM.tif]

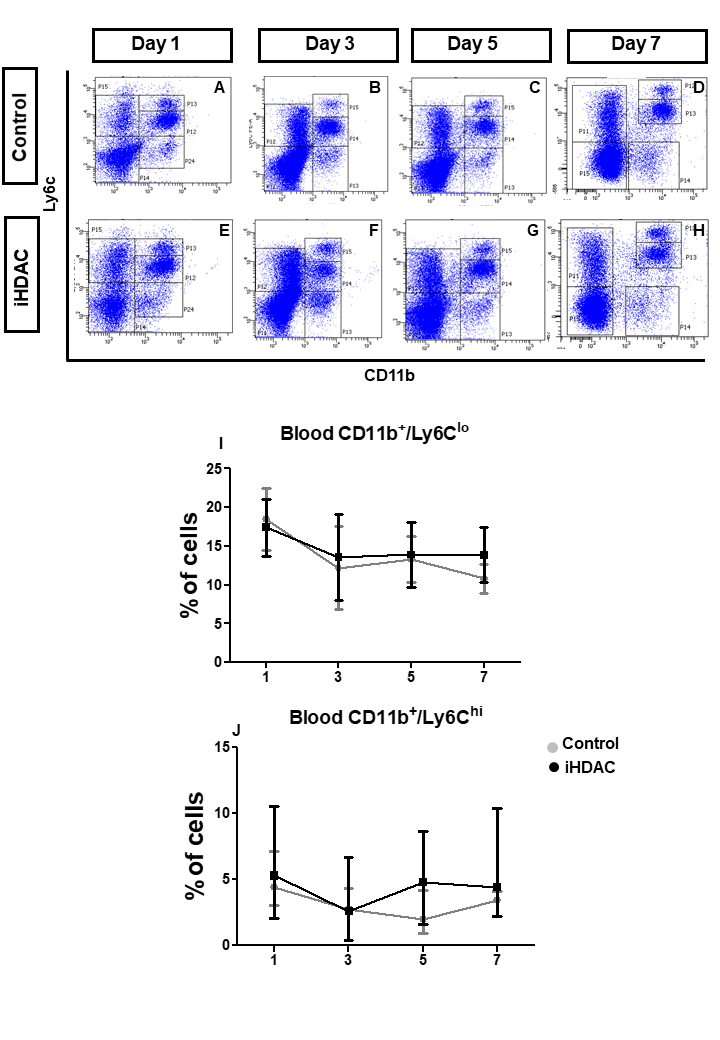

Supplement: Supplementary file 4 — Additional file 4: Figure S4. Topic iHDAC on wounds does not mobilize peripheral blood cells. Upon wound induction blood cells subsets were monitored by flow cytometry for 7 days (A–H) and any significant differences were detected in different blood subsets (I, J). N = 6. [file 12967_2019_1998_MOESM4_ESM.tif]

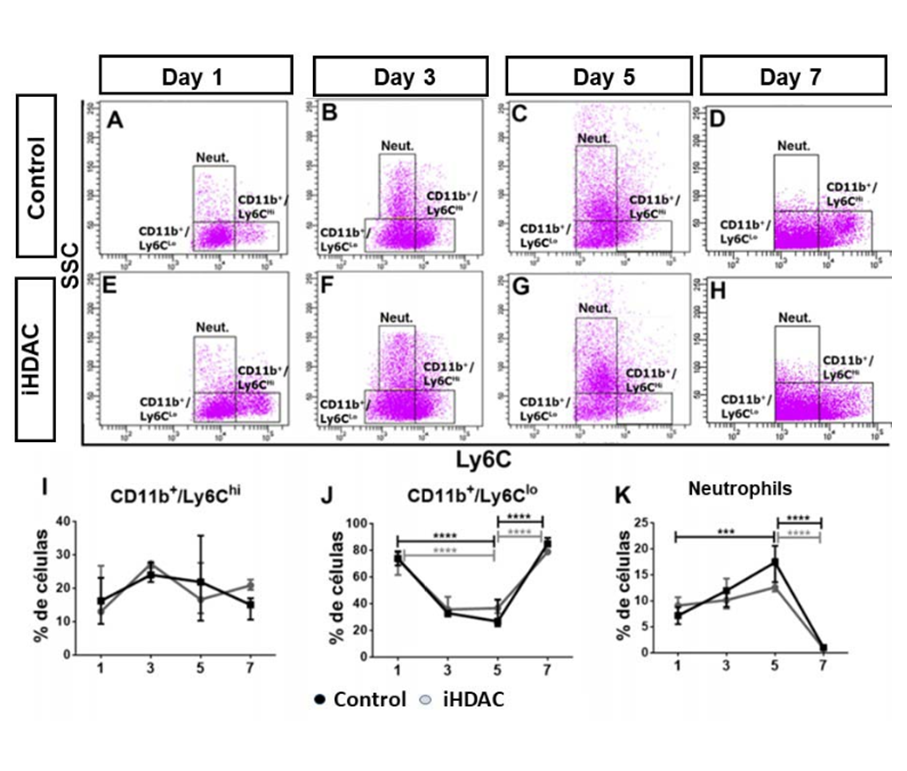

Supplement: Supplementary file 5 — Additional file 5: Figure S5. iHDAC does not alter peripheral blood subsets dynamics in wounds upon infiltration along the time. Upon wound induction, different subsets from peripheral blood infiltrated into the wounds were monitored for 5 days (A–F). Any significant difference was detected between control and iHDAC wounds with regard the infiltrating cells along the time (G–I). N = 6. [file 12967_2019_1998_MOESM5_ESM.tif]
